# Cardiometabolic Disease Risk Factors and Lifestyle Behaviors Among Adolescents: A Latent Class Analysis

**DOI:** 10.3390/healthcare13080925

**Published:** 2025-04-17

**Authors:** Fernanda Rocha de Faria, Valter Paulo Neves Miranda, Cheryl Howe, Jeffer Eidi Sasaki, Alessandra Amato, Giuseppe Musumeci, Paulo Roberto dos Santos Amorim

**Affiliations:** 1 Department of Physical Education, Federal University of Viçosa, Viçosa 36570-900, Brazil; frfaria.ef@gmail.com; 2 Federal Institute of Education, Science and Technology Goiano, Uruta 75790-000, Brazil; valter.miranda@ifgoiano.edu.br; 3 School of Applied Health Sciences and Wellness, Ohio University, Athens, OH 45701, USA; howec@ohio.edu; 4 Department of Kinesiology, University of Wisconsin-Madison, Madison, WI 53706, USA; jesasaki@wisc.edu; 5 Department of Biomedical and Biotechnological Sciences, Section of Anatomy, Histology and Movement Science, School of Medicine, University of Catania, 95123 Catania, Italy; alessandra.amato@unict.it (A.A.); g.musumeci@unict.it (G.M.); 6 Research Center on Motor Activities (CRAM), University of Catania, 95123 Catania, Italy

**Keywords:** risk factors for cardiometabolic disease, lifestyle, adolescents, latent class analysis

## Abstract

**Background/Objectives**: Cardiometabolic disease (CD) risk factors refer to the conditions that increase the likelihood of developing several health complications. The purpose of this study was to identify latent classes of CD risk factors among Brazilian adolescents and their association with sociodemographic and lifestyle behaviors. **Methods**: This was a cross-sectional study involving 349 adolescents aged 15 to 19 years old. A latent class analysis (LCA) was performed based on body mass index, body fat percentage, waist circumference, waist-to-height ratio, and blood pressure. Demographic characteristics and lifestyle variables related to screen time (ST), moderate-to-vigorous physical activity (MVPA), sedentary behavior (SB), and sleep duration were assessed through questionnaires. **Results**: Three CD risk factor classes were identified as follows: “Low Risk” (Class 1 = 79.5% of the sample), “Moderate Risk” (Class 2 = 8.6%), and “High Risk” (Class 3 = 11.9%). Sex and high ST (defined as >4 h/day) were associated with a greater likelihood of belonging to the higher CD risk classes. Adolescents with high ST presented a 4.39 (CI 95% 1.64–11.07) times greater chance of belonging to the “High Risk” instead of the “Low Risk” class. Adolescents with longer MVPA time had a higher probability of belonging to the “Low CD Risk” class. **Conclusions**: Female adolescents with less MVPA, more ST, and higher SB had a higher probability of being classified as “Higher CD Risk”. Efficient strategies to increase MVPA and reduce ST may contribute to the reduction in body fat accumulation and BP, which are the manifest variables in the proposed model.

## 1. Introduction

Cardiometabolic disease (CD) risk factors refer to health conditions that increase the likelihood of developing type 2 diabetes, vascular events [1,2], and other complications that can significantly impact the quality of life. Although the main symptoms typically manifest in adulthood [3], indicators such as abdominal obesity and high blood pressure (BP) can already be present in childhood [4] and adolescence [5]. In Brazil, population-based studies with adolescents have shown that 24.1% present borderline or elevated BP values [6], 25.5% are overweight or obese [6], and 12.6% exhibit high waist circumference (WC) [7], elevated body fat percentage (BF%) [8], or an increased waist-to-height ratio (WHtR) [9]. In addition, lifestyle-related behaviors, such as low physical activity levels [10,11], excessive sedentary behavior (SB) [12,13], and prolonged screen time (ST) [12,14], have also been strongly associated with increased cardiometabolic risk in this age group [15].

Traditionally, studies have examined the relationship between physical behaviors and CD risk factors using regression-based approaches, often adjusting for sociodemographic variables [16]. While useful, these methods tend to evaluate risk factors in isolation, potentially underestimating their combined effects and increasing the likelihood of type I errors [17]. This limitation stems from the fact that CD risk is a latent construct, that is, it reflects the interaction of multiple observable indicators rather than any single variable alone.

To address this complexity, some researchers have employed advanced statistical techniques, such as a latent class analysis (LCA), particularly in studies involving Brazilian adolescents [13,18]. LCA, a form of mixture modeling within the structural equation modeling framework, allows for the identification of unobserved (latent) subgroups within a population based on shared patterns of behavior or risk. This person-centered approach offers a more comprehensive view of how lifestyle factors cluster in adolescence, enabling the detection of high-risk profiles that might not be evident using traditional methods [13,19].

Despite its potential, a few studies have explored patterns or classes of CD risk factors, specifically in adolescents [18]. The most available research remains focused on metabolic syndrome components in adult populations [16], with limited investigation in adolescents [13,18,19]. Through LCA, however, researchers can classify individuals into latent classes based on sociodemographic, behavioral, and clinical characteristics. For instance, Miranda et al. [20] identified a latent class labeled “Inactive & Sedentary” among female adolescents, which was linked to excess body fat, insulin resistance, and elevated levels of high-sensitivity C-reactive protein—biomarkers that were further associated with increased concentrations of TNF-α, IL-6, and leptin. The risk factors in male adolescents appear to differ from those in females, likely due to boys being more physically active during adolescence [19]; however, this information is not clearly established.

These findings underscore the value of analyzing the co-occurrence and interaction of multiple risk factors within female and male adolescent subgroups. The use of LCA in this context enhances our understanding of how cardiometabolic risks are distributed across the adolescent population and supports the design of more precise, targeted interventions [17]. This approach has important implications for the development of public health policies and school-based strategies aimed at early prevention, especially for the most vulnerable groups. Therefore, the aim of this study was to identify latent classes of cardiometabolic disease risk factors among Brazilian adolescents, considering sex differences and their associations with sociodemographic and lifestyle variables. We hypothesize that after building the latent model, body composition measurements, together with blood pressure, will be identified as having the greatest interaction in characterizing the classes at higher risk for CD, as well as their association with other behavioral and sociodemographic variables.

## 2. Materials and Methods

### 2.1. Participants and Procedures

This was a cross-sectional study, comprising a random sample of adolescents enrolled in the technical high school of the Federal Institute of Education, Science, and Technology of Triângulo Mineiro (FIESTTM), Minas Gerais, Brazil. The study protocol was conducted according to the Declaration of Helsinki and was approved by the Research Ethics Committee involving human beings of the Federal University of Viçosa, under decision number 74104217.3.0000.5153. Before conducting any measures, all the adolescents provided written assents, while the parents or legal guardians of adolescents under 18 years provided written consent for participation in the study.

Eight Campi of FIESTTM offer high school courses along with technical education in six different cities. In general, 2653 adolescents (51.1% male) were enrolled in the institute with high school grades at the time of the sample selection. To calculate the sample size, we used a specific formula for cross-sectional studies contained in the EpiInfo software, version 7.2.2.16 (Atlanta, GA, USA). We set the population size at 2653 and the outcome prevalence at 50%, since the study considers multiple CD risk factors [20]. We adopted an acceptable error of 5% and a confidence level of 95%. The calculation indicated a minimum sample size of 336 adolescents. We increased the sample size by 10% (34 adolescents) to account for possible losses and used an effect design of 1.2, which indicated an ideal sample of 370 adolescents. The sample was recruited through simple random sampling. Adolescents were representative of the grade and sex of the students attending each campus.

To be included in the study, the adolescents were required to be between 15 and 18 years old, have returned the signed assent and consent forms, and be regularly enrolled in a high school grade of the institute. The exclusion criteria included pregnancy, temporary or permanent physical or mental disability, and the regular use of diuretics/laxatives or the use of medication to control BP.

The adolescents received a verbal description of the questionnaires before filling them out and were asked to answer honestly. The adolescents scheduled a specific day and time, according to their availability, to have their anthropometrics and resting BP measured using standard protocols [21]. The same trained evaluator performed all measurements throughout the study.

### 2.2. Cardiometabolic Risk Factor Variables

The adolescents’ weight (kg) and height (cm) were measured by a digital scale (Plenna^®^, São Paulo, Brazil) and a portable stadiometer (Sanny Medical^®^, São Paulo, Brazil), according to Lohman et al. [22]. The adolescents were barefoot, wore light clothes, and removed any ornaments. Their body mass index (BMI) was calculated through the standard formula (weight (kg)/height (m)^2^). BMI was classified by the z-score, according to sex and age [23].

BF% was estimated using an adipometer (Cescorf^®^, Porto Alegre, Brazil) and the equation proposed by Slaughter et al. [23]. The adolescents were told not to perform any type of physical activity for a minimum period of 4 h before the assessment. The tricipital and the medial calf skinfolds were obtained on the right side of the body, according to the protocol established by the International Society for the Advancement of Kinanthropometry [21]. The final values of the tricipital and the middle calf skinfolds were obtained from the average of three measures. BF% was classified according to Lohman [22], with values under 25% and 20% considered appropriate for females and males, respectively.

Waist circumference (WC) was measured in duplicate using a flexible and inelastic measuring tape (Cardiomed, Curitiba, Paraná, Brazil). The waist circumference was measured horizontally at the umbilical scar, reporting the average value of the three repeated measures. WC was classified as elevated CD risk if ≥90th percentile [24]. WHtR was calculated by dividing the average WC (cm) by height (cm). The value of WHtR ≥ 0.5 was considered an indicator of elevated CD risk [25].

BP was measured using an automatic device (Omron, model HEM 7200, Kioto, Japan). The measure was conducted according to the recommendations of the Guidelines of the Prevention of Atherosclerosis in Childhood and Adolescence [3]. The average of the last two of three measurements was considered as the real BP. The systolic and diastolic BP values were classified according to the age, sex, and height percentiles [3].

### 2.3. Lifestyle Behaviors

The participants self-reported lifestyle behaviors through paper questionnaires. To assess ST, the participants were asked the following: “On an average day, how many hours do you spend in front of any screen?”. The adolescents were told to consider all kinds of screens, such as mobile phones, TVs, computers, tablets, and video games. We chose to use the 25th percentile, which corresponded to >4 h, to classify them as either “appropriate ST” (<25th percentile) or “high ST” (defined as >4 h/day), according to the proposed classification of studies that have previously evaluated ST of Brazilian adolescent students [13,18,19].

Moderate-to-vigorous physical activity (MVPA) and sedentary behavior (SB) were assessed by the short version of the International Questionnaire of Physical Activity (IPAQ), previously validated for Brazilian adolescents [26]. MVPA was evaluated through sections 2 and 3 of the questionnaires. MVPA was considered adequate when the participants met 420 min per week, according to the recommendations of 60 min/day of MVPA [27,28]. The SB values on weekdays and weekend days were measured in sections 4a and 4b, respectively. We calculated the weighted means of these values to estimate the SB for the whole week. Due to the absence of a cutoff point for SB, the 75th percentile of the current dataset was used to classify the adolescents as “adequate SB” or “high SB” [13,19,29].

Sleep duration was assessed by the question: “On a normal day, how many hours do you sleep at night?”. The adolescents were instructed to disregard any sleep periods during the day. The average sleep duration between 8 and 10 h/day was classified as “adequate sleep” [30].

### 2.4. Covariates

Adolescents self-reported the sociodemographic characteristics of sex, age, grade, and socioeconomic status. Socioeconomic status was measured through a specific questionnaire proposed by the Brazilian Association of Survey Companies [31]. The questionnaire assigns different scores based on dwelling characteristics and the educational level of the household head. The adolescents’ socioeconomic status was divided into three categories based on the score reached: wealthy (classes A and B1), middle (classes B2 and C1), and lower class (classes C2 and D–E).

### 2.5. Statistical Analysis

Statistical analyses were conducted in the IBM Statistical Package for the Social Sciences (SPSS Statistics) (IBM Corporation, Armonk, NY, USA), version 21, and the R Statistical Software (R Development Core Team, 2014), version 3.2.2 (“Fire Safety”). The alpha level was set at 0.05 to interpret the test results.

The variables were categorized dichotomously to facilitate the interpretation of the results. A multiple correspondence analysis (MCA) was performed as a preliminary step to verify correspondence, dispersion, and approximation of the variables’ categories that represented the CD risk factors in the adolescents. This exploratory analysis and its graphical representation provided insight into the indicator variables and the number of latent classes for the model [13,18]. The distribution of the categories and their internal correlation coefficient were analyzed by the inertia value and Cronbach’s alpha for each dimension. After this analysis, BMI, BF%, WC, WHtR, and BP were selected as the manifest variables.

LCA was used for modeling the “CD Risk Factors” variable. This method is more appropriate for the analysis of interactions and associations between different kinds of latent variables. LCA was conducted in the poLCA package (polytomous variable latent class analysis) [32], available in the library of the R statistical software, version R-4.5.0 for Windows.

Diagnostic evaluation of the most parsimonious model—that which offers the best description of manifest variable observations for the fewest parameters estimated (which depends on the number of manifest variables and covariates)—was performed considering the Akaike Information Criterion (AIC), Bayesian Information Criterion (BIC), chi-squared goodness-of-fit test (χ^2^), and entropy. The model quality with the inclusion of the covariates was evaluated by likelihood ratio tests (G^2^). The selection of the final model also took into account the interpretability of the item-response probabilities of the manifest variables conditioned to the latent classes based on the homogeneity and separation of the classes.

The Kruskal–Wallis test was used to verify the association of the following covariates in the prevalence of the following classes: sex, age, socioeconomic status, sleep duration, MVPA, SB, and ST. Bonferroni’s correction was used in the two-by-two post hoc tests to verify the difference between the k groups. Effect sizes were calculated to evaluate the differences between the continuous values of SB and sleep duration among the three latent classes. The formulas for the statistical tests of Mann–Whitney U and Kruskal–Wallis H were used to calculate η^2^. The effect sizes were classified according to the cutoff points [33]. 

## 3. Results

Among the 394 adolescents who were invited to participate in the study, 19 declined the invitation, 6 were excluded for taking medication, and 8 were not between 15 and 18 years old. From the 361 who completed the survey, 12 were excluded due to missing information, resulting in an analytic sample of 349 adolescents. Most of the final sample was aged 15–16 years (66.19%), were male (51.58%), and belonged to the middle socioeconomic class (59.60%). The characteristics of the sample are reported in Supplementary Material S1.

**Figure 1:**
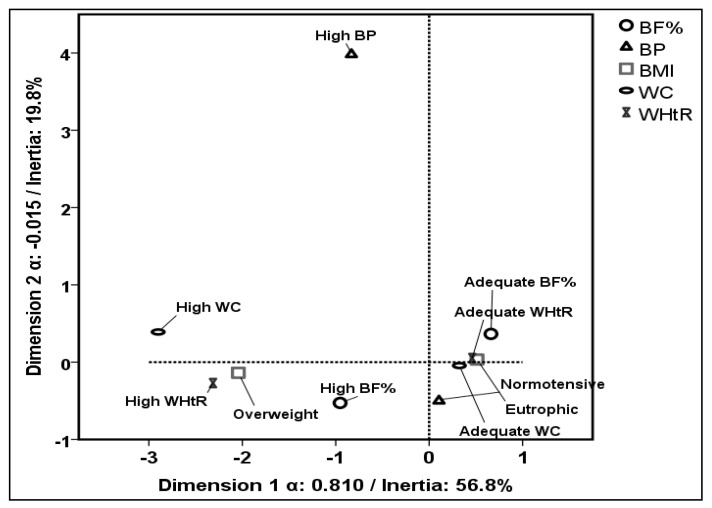
Multiple correspondence analysis of variables related to adolescents’ cardiometabolic disease risk factors. Cronbach’s α. BMI: body mass index; BP: blood pressure; BF%: body fat percentage; WC: waist circumference; WHtR: waist-to-height ratio.

Figure 1 shows the graphical representation of the association between different CD risk factors provided by the MCA. Dimensions 1 and 2 together explained 76.6% of the total variability. The internal correlation coefficients (Cronbach’s α) were 0.810 (high value) and 0.015, which means a moderate internal correlation.

After this exploratory step, the manifest variables BMI, BF%, WC, WHtR, and BP were chosen. Table 1 shows the model fit statistics from two to five latent class solutions. The model with three latent classes was the most appropriate for the data, presenting good values of absolute and relative model fit, parsimony, homogeneity, and separation of classes. In comparison with other models using different numbers of classes, the three-class model had similar metrics when compared to the model with two latent classes and better values compared to the others. In addition, the three-class model allowed for better interpretability of the item-response probabilities of the manifest variables associated with the CD risk factors.

**Figure 2:**
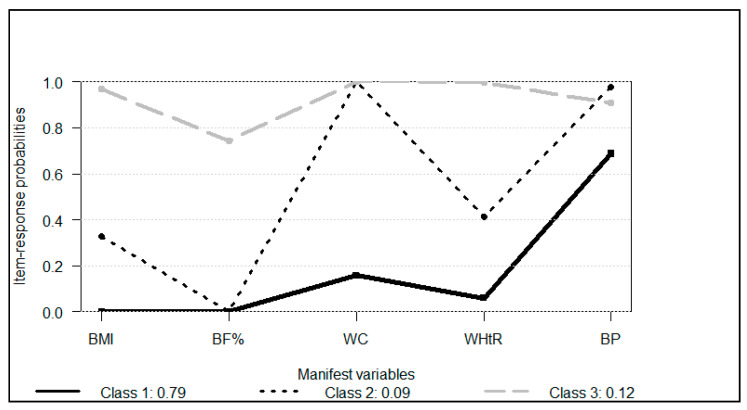
Profile plot of the LCA Model associated with Risk Factors of Cardiometabolic Diseases (CD) of adolescents. Class 1: ‘Low Risk of CD’, Class 2: ‘Moderate Risk of CD’, and Class 3: ‘High Risk of CD’. BMI: body mass index; BP: blood pressure; BF%: body fat percentage; WC: waist circumference; WHtR: waist-to-height ratio.

The three latent classes identified in the full sample are illustrated in Figure 2, and the selection of the three-class model proved to be the most adequate in representing the data. **Class 1**, labeled “Low Risk”, included 277 adolescents (γ = 79.5%) and was characterized by a high probability—above 90%—of presenting appropriate values for all of the manifest variables (BMI, WC, WHtR, and BP), except for body fat percentage (BF%), which showed slightly lower adequacy. **Class 2**, or “Moderate Risk”, consisted of 30 adolescents (γ = 8.6%) and stood out for its unfavorable profile in adiposity indicators; none of the adolescents had appropriate values for BF%, and only around 40% met the recommended values for BMI and WHtR. **Class 3**, identified as “High Risk”, included 42 adolescents (γ = 11.9%) and was marked by a generalized low probability (0–10%) of adequate values for BMI, BF%, waist circumference (WC), and WHtR, making these anthropometric indicators the most decisive in distinguishing this group from the Moderate Risk class. Additionally, blood pressure (BP) was consistently the variable with the lowest proportion of recommended values in both “Moderate” and “High Risk” classes, reinforcing its relevance as a marker of increased cardiometabolic vulnerability. The sharper contrast in central and total adiposity indicators (especially WC and WHtR) was key to differentiating Class 3 from Class 2, suggesting a progression in metabolic risk severity across the groups.

The covariates sex and ST were associated with the three-class model, as shown in Table 2. Female adolescents presented 10.28 (CI 95% 2.77–38.09) more chances of belonging to the “Moderate Risk” class (Class 2) instead of the “Low Risk” class (reference). Regarding the ST, it was found that adolescents with ST greater than 4 h/day presented 4.39 (CI 95% 1.64–11.07) times more chances of belonging to the “High Risk” class (Class 3) instead of the “Low Risk” class (reference). SB, despite not being significant (*p*-value = 0.062), pointed to a similar pattern. The adolescents with high SB were 2.20 (CI 95% 1.05–4.61) times more likely to belong to the “High Risk” class (Class 3) when compared to the “Low Risk” class (Class 1—reference).

Table 3 shows the variation of the values related to age and lifestyle behaviors among the three classes. The adolescents of the “High Risk” class (Class 3) had greater SB on the weekend than the adolescents of the “Low Risk” and “Moderate Risk” classes (Class 1 and Class 2). The adolescents of the “Low Risk” class (Class 1) had greater MVPA than the adolescents of the “Moderate Risk” class (Class 2). Additionally, the adolescents belonging to the “High Risk” class tended to have fewer hours of sleep (*p*-value = 0.060) and longer SB time during the whole week (*p*-value = 0.062) compared to the others in the “Low Risk” and “Moderate Risk CD” classes.

**Figure 3:**
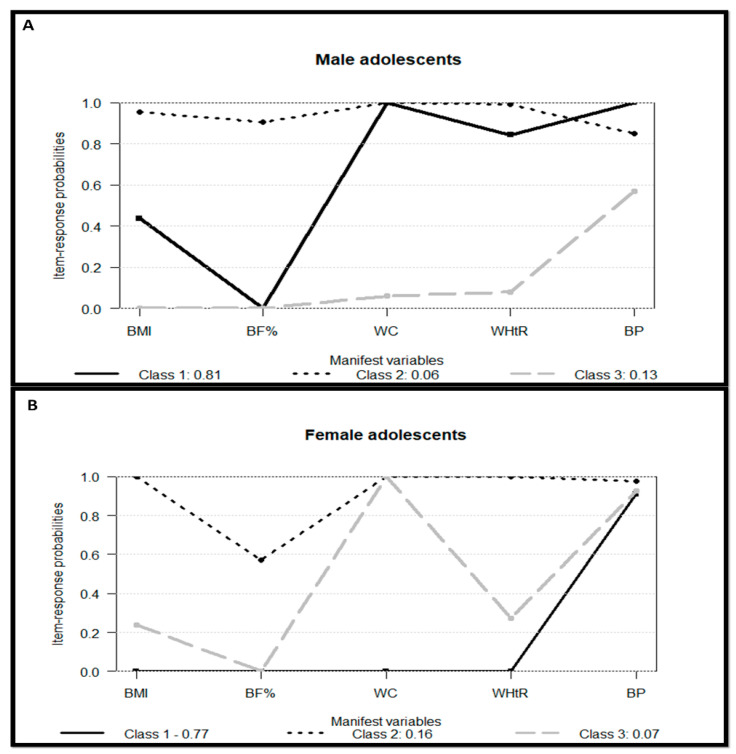
Profile plots of the latent class models associated with the cardiometabolic disease risk factors separately by sex. (**A**) AIC = 589.78; BIC = 644.04; χ^2^ = 10.56, Degree of Freedom = 14, G^2^ = 9.39 (*p*-value = 0.805), and entropy = 0.997. (**B**) AIC = 540.52; BIC = 593.73; χ^2^ = 1.38, Degree of Freedom = 14, G^2^ = 1.55 (*p*-value = 0.999), and entropy = 0.917. Class 1: “Low Risk”, Class 2: “Moderate Risk”, and Class 3: “High Risk”. Abbreviation: BMI: body mass index; BF%: body fat percentage; WC: waist circumference; WHtR: waist-to-height ratio; BP: blood pressure.

Regarding sex, the models showed that female adolescents had a higher prevalence in the “Low” and “Moderate” CD risk classes (23%) compared to male adolescents (19%). Figure 3 displays the three classes identified for each sex, jointly with adjustment values of absolute and relative model fit and entropy. The bar plot with the *p*-value of Fisher’s exact test is provided in Supplementary Material S2. There was a significant difference in the prevalence of the classes according to sex. Boys were more prevalent than girls in the “Low Risk” class (148 (42.4%) versus 132 (37.8%)) and in the “High Risk” class (25 (7.2%) versus 12 (3.4%)). Conversely, female adolescents had a higher prevalence in the “Moderate Risk” class (25 (7.2%) versus 7 (2.0%)). None of the covariates tested for the model with the whole sample were significantly associated with this model.

## 4. Discussion

We aimed to identify classes of adolescents based on the presence of CD risk factors and to evaluate demographic differences in the emergent classes. To our knowledge, this is the first study to apply LCA to a range of routine anthropometric- and clinical-based indexes to estimate CD risk factor classes among adolescents. The results found in this study will be helpful for public health in Brazil and other countries. The latent classes corresponding to CD risk were generated from the interaction of different characteristic factors of the studied adolescent sample. It was found that BMI, BF%, WC, WHtR, and BP, when analyzed simultaneously, resulted in an adjusted model with three types of CD risk classification for students at a Brazilian federal institution.

Overall, approximately 72 adolescents (21%) presented three or more risk factors (a sum of Class 2 and Class 3). A key observation from the analysis is that patterns of CD risk vary among adolescents based on sex and ST. Further, there is a difference in lifestyle behaviors (i.e., physical activity and SB) among the classes. Identifying classes of cardiometabolic risk factors in adolescents is essential to find out who is at increased risk for developing CD and to tailor efficient interventions. It makes an important contribution to the literature by investigating the co-occurrence of traditional cardiometabolic risk factors during adolescence.

We did not find the best possible class in which all the adolescents had adequate values for all the manifest variables included in the LCA. Even Class 1, considering the “Low Risk” class (n = 277), had a little more than 20% of the adolescents with high values of BF%. As well established in the literature, high BF% is associated with hypertension, dyslipidemia, and type 2 diabetes mellitus [8]. Despite that, more than 90% in this class showed adequate values for the other four risk factors evaluated. We also highlight that the “Low Risk” class had greater MVPA compared to the “Moderate Risk” class. Our results are supported by previous research that has shown the inverse relationship between physical activity and BMI [34], BF% [35], and WHtR [36]—the three main variables that distinguished both classes. However, although the “Low Risk” class (Class 1) had better values of MVPA per week (median: 180 min/week), it was still below the current recommendation of 420 min per week of MVPA [20,28]. Future interventions should promote MVPA in this class and follow its effects on cardiometabolic risk factors, mainly on BF%.

All of the adolescents belonging to the “Moderate Risk” class (Class 2 = 30 adolescents) had high values of BF% as a common feature. Moreover, girls were more likely to be in this class than in the “Low Risk” class (Class 1). These facts are consistent with the literature, which has shown a greater prevalence of high BF% among girls when compared to boys [37]. Additionally, 60% of the adolescents in this class were more likely to have high values of BMI and WHtR. BMI is associated with many mortality and comorbidities, such as type 2 diabetes mellitus, coronary heart disease, and ischemic stroke [38]; meanwhile, WHtR is a measure of central obesity [39], known as an efficient index for predicting CD risk. Interventions tailored to this class should help to reduce their adiposity values by reducing SB and promoting physical activity of any intensity. It is important to highlight that this class was predominantly composed of girls, and gaining a deeper understanding of the unique characteristics and preferences of this group may contribute to the development of more effective interventions.

The “High Risk” class (Class 3), the worst class found, included 42 adolescents with concerning health conditions, most of them presenting with at least four cardiometabolic risk factors. All of the adolescents in this class had elevated BMI and BF%, and more than 80% had high values of WC and WHtR. The prevalence of this class was affected by ST. The adolescents with more than 4 h/day of ST were more likely to belong to this class than to the “Low Risk” class (Class 1). According to the literature, adolescents should not spend more than 2 h/day on ST [40]. However, less than 5% of our sample achieved this cutoff. We highlight that the technology revolution, which has been occurring in the past few years, has popularized and introduced new electronic devices with different apps and functions (such as smartphones), and this has made it difficult to keep within the 2 h/day for ST. For our sample, 4 h/day was the cutoff associated with the worst class, similar to the results of other studies that evaluated ST among Brazilian adolescents from the same sociodemographic context [13,18,19,29]. In the study by Miranda et al. [20], it was found that adolescents belonging to the class with a less active and more sedentary lifestyle were associated with risk factors for cardiometabolic diseases. Our results corroborate with the study developed by Rey-Lopez et al. [41], in which a sample of 2200 adolescents had their physical activity and ST self-reported. It was found that 4 h/day of ST based on time spent watching TV was a significant predictor of the probability of being obese after adjusting for physical activity [41]. These studies are not alone in pointing out that most adolescents exceed the 2 h/day limit of ST [13,42]. For the recommendation to be followed, it must be reachable. In this context, future studies should look for the applicability of this recommendation, considering the new technology era we live in, as well as all the changes triggered by the recent COVID-19 pandemic, including the educational process, which has led to an increase in screen time.

The “High Risk” class (Class 3) also showed a higher SB on weekend days (median = 12 h/day) compared to the other classes. This result is consistent with the adolescents’ weekly routine. Adolescents participating in this study attend high school classes along with technical education every weekday, from 7:30 am to 4:50 pm. That is, during the weekday, their routine is similar for most of their daytime, with the predominance of activities based on SB. During the weekend, they do not have a strict schedule to follow and are free to participate in different types and intensities of activities. The difference in physical activity and SB patterns between weekdays and weekend days has been previously reported [10]. This difference should apply to adolescents enrolled in full-time school. We speculate that if the International Physical Activity Questionnaire (IPAQ) measured physical activity according to week and weekend days, it would be possible to find a greater MVPA on the weekend. Interventions targeting this class should not only promote changes in lifestyle behaviors (such as MVPA and SB) but also make them aware of their health risks during adolescence and adulthood.

Overall, the results found for the “Moderate Risk” and “High Risk” classes should not be overlooked. The harmful health conditions presented in these classes are a public health concern, since these conditions tend to track into adulthood. Promoting healthy choices is a key factor in the fight against cardiometabolic risk factors, mainly in adolescents. As such, schools appear as an appropriate place to promote this type of knowledge. The intervention potential is even greater in full-time schools. Adolescents enrolled in this type of school spend more of their waking time in this environment than with their families. Therefore, interventions in this environment should focus on identifying who is at high risk, their living conditions, and lifestyle to tailor strategies to their needs to foment the adoption of healthy choices.

Male adolescents were more prevalent in the extreme classes (“Low Risk” and “High Risk”), while females were more frequent in the intermediate class (“Moderate Risk”). The cardiometabolic profile for each sex revealed similar trends for the “Low Risk” and “High Risk” classes. For the “Low Risk” class, the main difference between sexes was related to BF%. Girls presented a greater prevalence of excess BF% compared to the boys. Meanwhile, males had a higher prevalence of high BP compared to females in the “High Risk” class. The “Moderate Risk” class was the most different between the sexes. For boys, the two most concerning risk factors were BMI and BF%, and for girls, it was WHtR and BP. This detailing per sex can provide helpful sources for the implementation of strategies to control cardiometabolic risk factors according to the preference of each sex. Addressing physical activity is one of the aspects related to the prevention of CD risk factors among adolescents. James et al. [43] showed a gap between what types of activities are provided and what adolescents want to do. A key finding in this study was the different qualities valued by girls in comparison to boys. The enjoyment and socialization aspects of the activity play an important role in girls’ engagement, which is not true for boys. Together, these findings reinforce the need for taking into account the preferences of boys and girls in the development of successful interventions.

The findings of this study, which highlight the negative impact of high ST and low levels of MVPA, underscore the need for translating evidence into actionable strategies. A study using an isotemporal analysis with a sample of over 5000 Korean children and adolescents showed that one-hour increments of any non-screen time activities, combined with a simultaneous one-hour decrease in total screen time, were associated with a lower prevalence of obesity in 25% (0.75–95%CI: 0.65, 0.85) [44]. Recently, a new federal law (Lei nº 15.100/2025) [45] in Brazil restricted the use of smartphones in elementary and high schools, allowing their use only in emergencies, for educational purposes, or by students with special needs. This measure reflects the growing concern about the negative impacts of excessive screen use on students’ mental health and academic performance. Efficient strategies to increase MVPA and reduce ST may contribute to the reduction in body fat accumulation and BP, which are the manifest variables in the proposed model.

The strengths of this study include its originality, a representative sample from six different cities in Brazil representing different environmental conditions, and the application of LCA to identify classes of CD risk factors based on BMI, BF%, WC, WHtR, and BP. We highlight that the model was based on measures that were easy to perform and with low cost, a methodology that can be replicated regardless of the financial limitation, to estimate profiles of CD risk factors, increasing ecological validity. However, this study has some limitations. This study has a cross-sectional design and cannot inform about the development and determinants of these patterns. Additionally, part of the variables were based on self-report, which is subject to bias. Due to methodology limitations, PA was assessed as the total duration of MVPA, regardless of the frequency. In addition, parents’ health history could have helped to explain the resulting adolescent cardiometabolic profiles.

## 5. Conclusions

In conclusion, cardiometabolic risk factors were classified into three latent classes. Approximately 21% of adolescents presented three or more CD risk factors. The prevalence of the classes was affected by sex and high ST. The “Low Risk” class had a greater time spent in MVPA compared to the “Moderate Risk” class, whereas the “High Risk” class had a higher SB time on weekend days compared to the other classes. Girls were more likely to belong to the “Moderate Risk” class, while boys were more prevalent in the other two classes. Our findings may help to tailor successful interventions specific to sex and risk level to combat CD risk factors in adolescence, helping to worsen these during adulthood.

## Figures and Tables

**Table 1:** Model fit indexes for latent class models.

Number of Classes	AIC	BIC	DF	χ^2^	G^2^	p-G^2^	Entropy
2 Classes	1194.32	1236.73	11	29.01	32.24	0.04	0.91
3 Classes *	1186.06	1251.59	14	17.10	11.97	0.61	0.88
4 Classes	1191.25	1279.91	8	9.86	5.17	0.74	0.93
5 Classes	1198.22	1310.02	2	0.08	0.15	0.93	0.78

* Selected model. Abbreviations: AIC: Akaike Information Criterion; BIC: Bayesian Information Criterion (BIC); DF: Degree of Freedom; χ^2^: Pearson’s Goodness-of-Fit; G^2^: Likelihood Ratio Deviance Statistic; p-G^2^: *p*-value of Likelihood Ratio Deviance Statistic.

**Table 2:** Sex, screen time, and sedentary behavior as predictors of membership prevalence in latent classes associated with cardiometabolic disease risk factors in adolescents.

**α (Intercept)**	**Moderate Risk/Low Risk**					
	**β** **(Coefficient)**	**SE**	**Odds Ratio**	**CI (95%)**		***p*-Value**
Female ^†^	2.33	0.67	10.28	2.77	38.09	0.004 **
High ST (25th P) ^‡^	0.07	0.27	1.07	0.63	1.82	0.790
High SB ^¥^	−1.06	1.32	0.34	0.02	4.61	0.439
	**High Risk/Low Risk**					
	**β (Coefficient)**	**SE**	**Odds Ratio**	**CI (95%)**		***p*-Value**
Female ^†^	0.55	0.37	1.74	0.84	3.59	0.186
High ST (25th P) ^‡^	1.48	0.50	4.39	1.64	11.07	0.013 *
High SB ^¥^	0.79	0.38	2.20	1.05	4.61	0.062

^†^ Indicates that “male” is the reference category. ^‡^ Indicates that “Adequate ST (under the 25th P)” is the reference category. ^¥^ Indicates that “Adequate SB (under the 75th P)” is the reference category. * Significant association for *p* < 0.05; ** Significant association for *p* < 0.01. Abbreviations: ST: screen time; SB: sedentary behavior; 25th P: 25th percentile; 75th P: 75th percentile. β: beta coefficient value; SE: standard error; CI 95%: confidence interval of 95%.

**Table 3:** Age and lifestyle behaviors according to the latent classes associated with the cardiometabolic disease risk factors among adolescents.

Variables	“Low Risk” Class		“Moderate Risk” Class		“High Risk” Class		*p*-Value
	Median	P25–P75	Median	P25–P75	Median	P25–P75	
Age (years)	16	15–17	16	15–17	16	15–17	0.886
MVPA (h/week)	3 ^††^	1–6	1.5 ^††^	0.17–3.7	2	0.2–4.5	0.014 *
SB—Week days (h/day)	11	9.5–13	10.5	9–12.5	12	9.3–14.5	0.402
SB—Weekend days (h/day)	9 ^†^	6–12	8 ^‡^	5.13–10.75	12 ^†‡^	9.2–14.5	0.002 **
SB 7 days (h)	10.4	8.6–12.6	10	7.8–12.1	11.4	9.4–15.1	0.067 ^£^
Sleep Duration (h/day)	7	6–7.5	6.5	5.12–7	6.5	6–7	0.060 ^¥^
ST (h/day)	6	4–9	6	4.6–11.5	6	5–10.12	0.112

* Significant *p*-value (<0.05), ** Significant *p*-value (<0.01) of the Kruskal–Wallis test. ^†^ Significant *p*-value (<0.016) of Bonferroni’s post hoc test between Class 1 and Class 3; ^‡^ Significant *p*-value (<0.016) of Bonferroni’s post hoc test between Class 2 and Class 3; ^††^ Significant *p*-value (<0.016) of Bonferroni’s post hoc test between Class 1 and Class 2; ^¥^ dCohen: 0.206 (small effect size [33]); ^£^ dCohen: 0.199 (small effect size [33]). Abbreviations: h: hour; MVPA: moderate-to-vigorous physical activity; SB: sedentary behavior; ST: screen time.

## Data Availability

The data underlying this article will be shared on request to the corresponding author.

## Supplementary Materials

The following supporting information can be downloaded at https://www.mdpi.com/article/10.3390/healthcare13080925/s1, Table S1: Association values of the Latent Class Analysis Model with individual. Figure S1: Prevalence of the latent classes that represent the Risk Factors of Cardiometabolic Disease in relation to sex. Viçosa-MG, Brazil.

## Abbreviations

The following abbreviations are used in this manuscript:

CD	Cardiometabolic disease
WC	Waist circumference
BF%	Body fat percentage
WtHR	Waist-to-height ratio
SB	Sedentary behavior
ST	Screen time
LCA	Latent class analysis
FIESTTM	Federal Institute of Education, Science, and Technology of Triângulo Mineiro
BP	Blood pressure
BMI	Body mass index
MVPA	Moderate-to-vigorous physical activity
AIC	Akaike Information Criterion
BIC	Bayesian Information Criterion
DF	Degree of Freedom
χ2	Pearson’s Goodness-of-Fit
G2	Likelihood Ratio Deviance Statistic
