# Supplementary material for: Cardiometabolic Disease Risk Factors and Lifestyle Behaviors Among Adolescents: A Latent Class Analysis
Source: Healthcare (Basel). 2025 Apr 17;13(8):925. doi: 10.3390/healthcare13080925 (PMC12026998; doi:10.3390/healthcare13080925)
Supplement: Supplementary file 1 [file healthcare-13-00925-s001.zip › Supplementary Material S1.pdf]

Supplementary file S1

Table S1. Association values of the Latent Class Analysis Model with individual covariates.

	Class 2 / Class 1‡						Class 3 / Class 1‡					
**Covariates**	**β (Coefficient)**	**SE**	**Odds Ratio**	**CI (95%)**		**p-value**	**β (Coefficient)**	**SE**	**Odds Ratio**	**CI (95%)**		**p-value**
Male†			1						1			
Female	1.09	0.71	2.97	0.74	11.94	0.150	1.97	0.46	7.17	2.91	17.63	0.001*
Without CMD†			1						1			
Presence of CMD	0.99	0.36	2.69	1.33	5.41	0.019*	2.37	1.09	2.97	1.27	90.01	0.05*
Eutrophic†			1						1			
Overweigth	-0.49	0.44	0.61	0.25	1.44	0.280	0.72	0.51	2.05	0.75	5.58	0.182
Adequate WHtR†			1						1			
Elevate WHtR	1.70	1.26	5.47	0.46	64.07	0.202	0.34	0.39	1.40	0.65	3.00	0.399
Normal BP†			1						1			
Elevated BP	-1.22	0.54	0.29	0.10	0.84	0.044*	-0.72	0.51	0.48	0.76	5.52	0.182
Alcohol - not exposed†			1						1			
Alcohol - exposed	0.30	0.50	1.34	0.50	3.59	0.550	-0.20	0.53	0.81	0.30	2.20	0.712
Tobacco - not exposed†			1						1			
Tobacco - exposed	0.28	0.64	2.24	0.37	6.61	0.664	-0.87	0.44	0.41	0.17	0.99	0.072
Adequate SD†			1						1			
Insuficcient SD	2.06	0.77	7.84	1.75	35.16	0.021*	0.52	1.25	1.68	0.14	19.49	0.682

‡Reference class, Class 1; †Reference categories; *Significative association (p-value ≤0.05).
Class 1: Active & Non-Sedentary; Class 2: Inactive & Non-Sedentary; Class 3: Inactive & Sedentary. CMD, Common Mental Disorder; WHtR, Waist-Height Ratio; BP, Blood Pressure; SD, Sleep duration.
