# Supplementary material for: Cardiometabolic Disease Risk Factors and Lifestyle Behaviors Among Adolescents: A Latent Class Analysis
Source: Healthcare (Basel). 2025 Apr 17;13(8):925. doi: 10.3390/healthcare13080925 (PMC12026998; doi:10.3390/healthcare13080925)
Supplement: Supplementary file 1 [file healthcare-13-00925-s001.zip › Supplementary Material S2.pdf]

Supplementary material S2: Bar plot with p-value of Fisher's Exact Test is presented as supplementary material 2

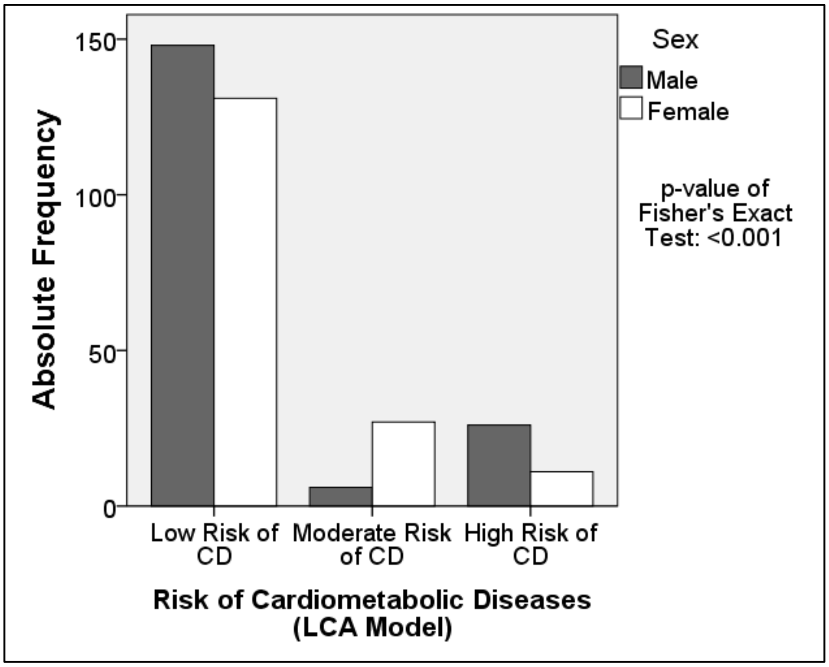

Figure S1: Prevalence of the latent classes that represent the Risk Factors of Cardiometabolic Disease in relation to sex. Viçosa-MG, Brazil.
